# The Morphine Habit as a Legal Defense

**Published:** 1898-08

**Authors:** 


### THE MORPHINE HABIT AS A LEGAL DEFENSE.

We take the following item from the July *Journal of Inebriety:*

"A kleptomaniac in one of the British courts plead guilty, and her counsel assured the bench that she was in no want of money, but had sufficient means to enable her to live comfortably, and asked that she be treated leniently on the ground that the theft was due to the effects of the excessive use of morphine. According to the testimony, she had consumed ninety-six grains of morphine in a single week. The magistrate suspended judgment, upon the defendant giving security in £50 to appear for sentence when required. De Quincey's daily consumption of laudannm was nine ounces, and there is a case on record where 120 grains of opium was taken at once without producing death. The tolerance of opium and its salts proves in reality much more than old women's fables, and instances of enormous doses are in the possession of nearly every family practitioner. A poisonous draught of laudanum can not be measured by cases on record.

